# Spectroscopic Studies of Copper and Silver Binding to Metallothioneins

**DOI:** 10.1155/MBD.1999.277

**Published:** 1999

**Authors:** Martin J. Stillman

**Affiliations:** Department of Chemistry The University of Westem Ontario ON London N6A 5B7 Canada

## Abstract

Mammalian metallothionein is remarkable in its metal binding properties: well-characterized species exist for metal to sulfur ratios of M_7_S_20_, M_12_S_20_, and M_18_S_20_, where M = Cd(ll), Zn(ll), Hg(ll), Ag(I), Au(I), and Cu(I). Circular dichroism and luminescence spectra provide rich details of a complicated metal binding chemistry when metals are added directly to the metal free- or zinc-containing protein. CD spectral data unambiguously identify key metal to protein stoichiometric ratios that result in well-defined structures. Emission spectra in the 450-750 nm region have been reported for metallothioneins containing Ag(I), Au(I), and Cu(I). The luminescence of Cu-MT can also be detected directly from mammalian and yeast cells. Qualitative and quantitative interpretations show that the final structure adopted by Ag-MT is not the same as that formed by Cu(I) ions in Cu-MT. XAFS structural data are reported for a number of metallothioneins, including Ag_12_-MT and Ag_17_-MT. Electrospray ionization mass spectrometry provides details on the species formed when Ag(I) binds to metallothionein. Mass spectral data are reported for metal-free MT 2A and Ag_n_-MT (n = 14-18).


### SPECTROSCOPIC STUDIES OF COPPER AND SILVER BINDING TO METALLOTHIONEINS

Martin J. Stillman

Department of Chemistry, The University of Westem Ontario, London, ON, N6A 5B7 Canada
e-mail: Martin.Stillman@uwo.ca

### ABSTRACT

Mammalian metallothionein is remarkable in its metal binding properties: well-characterized species exist for metal to sulfur ratios of $M_7S_{20}$, $M_{12}S_{20}$, and $M_{18}S_{20}$, where M = Cd(II), Zn(II), Hg(II), Ag(I), Au(I), and Cu(I). Circular dichroism and luminescence spectra provide rich details of a complicated metal binding chemistry when metals are added directly to the metal free- or zinc-containing protein. CD spectral data unambiguously identify key metal to protein stoichiometric ratios that result in well-defined structures. Emission spectra in the 450-750 nm region have been reported for metallothioneins containing Ag(I), Au(I), and Cu(I). The luminescence of Cu-MT can also be detected directly from mammalian and yeast cells. Qualitative and quantitative interpretations show that the final structure adopted by Ag-MT is not the same as that formed by Cu(I) ions in Cu-MT. XAFS structural data are reported for a number of metallothioneins, including $Ag_{12}$-MT and $Ag_{17}$-MT. Electrospray ionization mass spectrometry provides details on the species formed when Ag(I) binds to metallothionein. Mass spectral data are reported for metal-free MT 2A and $Ag_n$-MT (n = 14-18).

### INTRODUCTION

The metallothioneins (MT) are a class of metalloprotein characterized by an amino acid sequence that is unique in its high fractional content of cysteine and lack of aromatic residues[1-6]. The metal binding properties of the class 1 mammalian metallothioneins, especially those of the rabbit liver protein, have been widely studied[1-33]. The key remarkable property of the metallothioneins is their ability to bind an extensive range of metals both in vivo and in vitro, typically Zn, Cu, Cd and Hg, but also many others including Ag and Au, especially in vitro[6,8-12]. Rabbit liver MT contains 20 cysteines (as RSH) as part of a 61 (isoform 1) or 62 (isoform 2A) amino acid sequence[7], Figure 1. The absence of aromatic amino acids is a characteristic property of metallothioneins that allows analysis of the metal binding properties by optical techniques because the thiolate to metal charge transfer transitions in the 220 to 350 nm region would normally be completely masked by the presence of aromatic groups. Structural data for the metallothioneins comes from a variety of sources, but currently, mainly, NMR ($^{111,113}Cd$ and $^{1}H$), X-ray absorption spectroscopy (XAS) and X-ray diffraction[1-6,8-13]. The XAS experiments (particularly, extended X-ray absorption fine structure (XAFS) spectroscopy) have provided detailed metal-sulfur bond length information that allows structures to be proposed for metallothioneins that do not include the NMR-active nuclei[6].

We have used the fine detail associated with optical measurements, particularly UV-region circular dichroism and absorption, and visible-region emission spectroscopy[6] to characterize a number of different metal-thiolate clusters that form in the protein, including $Zn_7$-MT, $Cd_7$-MT, $Cu_{12}$-MT, $Hg_7$-MT, $Hg_{18}$-MT, $Ag_{12}$-MT, and $Ag_{17}$-MT[6,14-31].

Figure 1 shows the primary amino acid sequence for isoform 2A of rabbit liver metallothionein[7]. The mammalian protein comprises two metal binding domains that involve different clusters of metals, Figures 2 and 3. The N-terminal domain of the mammalian peptide is named β and binds the tetrahedrally-co-ordinated Zn(II) and Cd(II) metal ions in a cluster with stoichiometry of $M_3S_9$. The C-terminal domain of the peptide is named α in which the $M_4S_{11}$ cluster forms with Zn(II) and Cd(II). Figure 2 shows the metal-thiolate connectivities in the two metal binding domains as determined by NMR and X-ray diffraction techniques for Cd(II) and Zn(II). Using the $Zn_4S_{11}$ and $Zn_3S_9$ cluster structures shown in Figure 2, a space-filling representation of the three-dimensional structure of rabbit liver $Zn_7$-MT 2A was calculated with molecular modeling techniques[32], Figure 3.

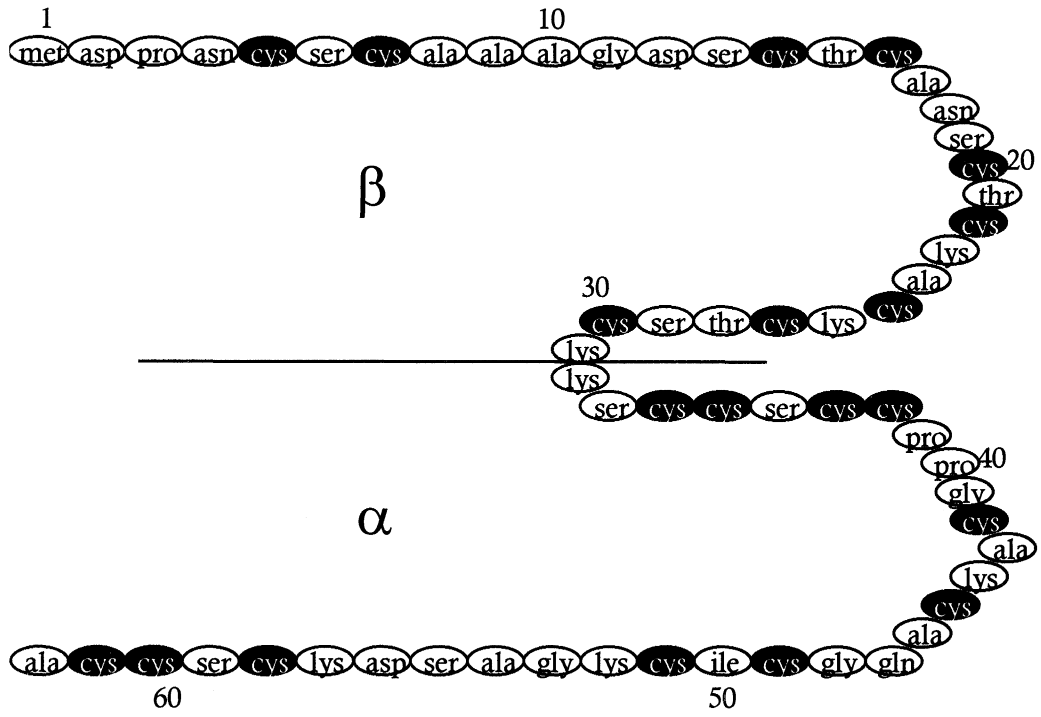

Figure 1 The sequences for rabbit liver metallothionein isoform 2A, where the dark ovals represent cysteine residues, based on data of Kagi[7]. The N-terminal forms the β domain, the C-terminal the α domain for metal binding. Metal binding takes place through the cysteinyl sulfurs. The metal-sulfur connectivities have been determined from analysis of X-ray diffraction studies and NMR studies (see refs 1-5).

What is clear from this description of the structure is that the three dimensional structure of the MT peptide chain is almost completely determined by the network of metal-thiolate bonds that form when the protein binds metals. The two metal-binding domains are three-dimensional structures, around which the peptide chain wraps. Because chiral structures are formed in this manner, the CD spectroscopic properties are sensitive to the wrapping, which is itself completely controlled by the number of metal-thiolate bonds formed. By monitoring titrations of the protein with metals using CD techniques we can observe formation of complexes with defined metal to protein stoichiometric ratios. The key to the information provided by this experiment is that the CD spectrum is dependent on two independent properties, (i) the presence of the chiral electric field induced in the binding site region by the wrapping of the peptide chain, and (ii) the energy of the sulfur to metal charge transfer transition. For (i), the intensity is controlled by the structure of the metal-thiolate structure itself, as the number of bound metals changes and as the coordination geometry changes (for example, from tetrahedral to trigonal) so the peptide wrapping changes and the CD band intensity and sign will change in tandem. For (ii), we find a chemical shift in the band maximum (a red or blue wavelength shift of the CD bands) that is dependent on the metal (here, Zn, Cd, Hg, for example) and the coordination geometry adopted. Thus the CD spectrum is uniquely sensitive to the formation and change of the metal-thiolate binding in the binding site itself. As we show below, silver metallothionein exhibits a characteristic CD spectral envelope that changes as a function of the silver loading, from 1 to 20.

To summarize, the properties of the mammalian protein are:

- a primary sequence that is dominated by a 30% cysteine content
- a molecular mass near 6,200 daltons (for the metal free mammalian protein)
- a complete absence of aromatic amino acids in the primary sequence
- a metal-thiolate clustered binding site that involves both bridging and terminal thiolate groups
- an absence of disulfide bonds which means that the three-dimensional structure of metal-free metallothionein is essentially that of a random chain

- a tertiary structure that is dominated by metal-thiolate clusters, with one domain for the yeast or fungus proteins and two domains for mammalian and crustacean proteins

- binding metals in vitro as diverse as Ag(I), Au(I), Bi(III), Cd(II), Co(II), Cu(I), Fe(II), Hg(II), Pb(II), Pt(II), Tc(IV), and Zn(II)

Questions to be answered in work to elucidate the structure and function of silver in metallothionein, or more generally, any metal and its interaction with a metalloprotein, include:

- To what extent is the metallothionein involved in the transport and homeostasis of the silver?
- Is silver unique in its metal binding properties when compared with other metals, for example, copper?
- How many silver atoms bind per protein molecule?
- What is the co-ordination geometry around each silver atom?
- How does the peptide chain wrap round the silver-thiolate clusters - is the wrapping the same as with other similar metals?

While, the data described here provide answers to only parts of these questions (the structural and stoichiometric aspects), the results illustrate that these techniques can be used in future studies to provide detailed information for each of the questions posed above. For example, the emission spectrum of Ag-MT[27] can be used to determine the presence of Ag-MT in the cell directly. The similarities between Cu(I) and Ag(I) binding to metallothioneins have been mentioned in many reports, in this paper, spectroscopic data that illustrate the similarities and differences between the binding of these two metals will be described.

### ISOLATION AND PURITY

$Zn_7$-MT was isolated from rabbit livers following *in vivo* induction procedures using aqueous zinc salts. The protein was purified using gel filtration and electrophoresis[17,30] or ion exchange chromatography[33] as previously described. Aqueous protein solutions were prepared by dissolving the lyophilized protein in argon-saturated deionized water. Protein concentrations were estimated from measurements of the -SH group and zinc concentrations. These estimates were based on the assumption that there are 20 -SH groups and 7 Zn atoms in each protein molecule.

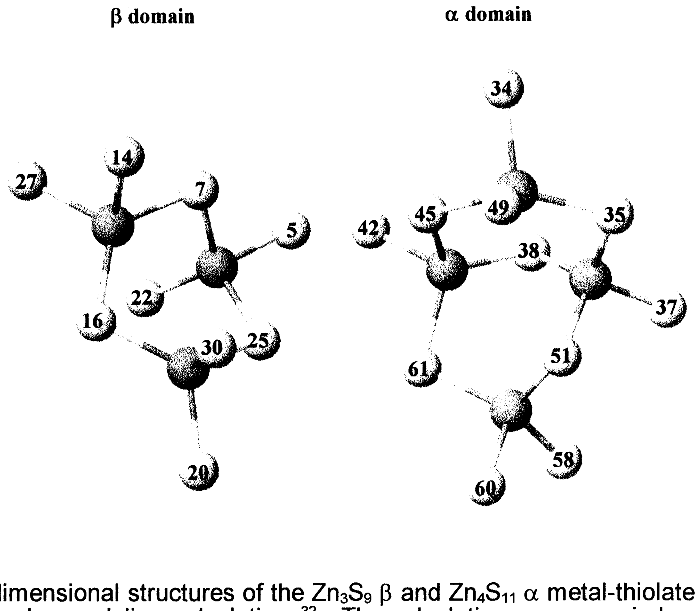

Figure 2 The three-dimensional structures of the $Zn_3S_9$ β and $Zn_4S_{11}$ α metal-thiolate binding clusters based on results from molecular modeling calculations[32]. The calculations were carried out using both molecular mechanics (MM2) and molecular dynamics techniques, see Figure 3. The dark spheres represent Zn atoms, the light spheres represent S atoms of cysteine residues in the peptide chain; the numbering corresponds to the cysteine residue in the rabbit liver MT isoform 2A sequence shown in Figure 1. Reproduced with permission from ref. 7.

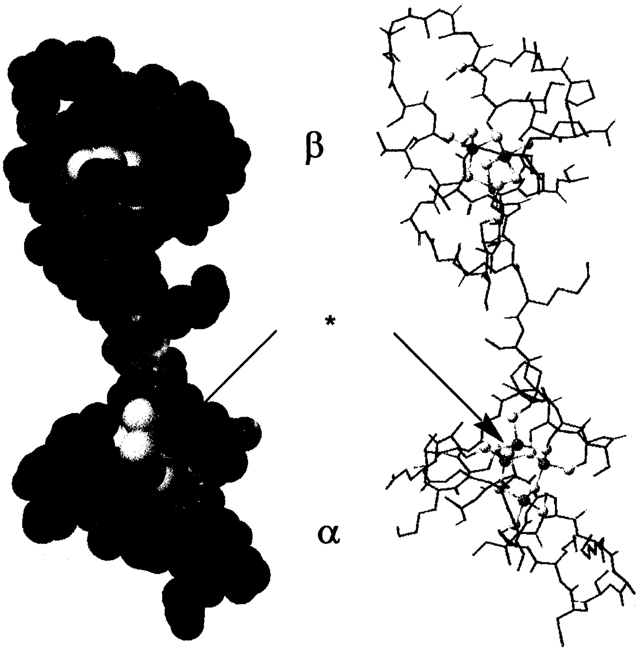

*** Denotes accessible sites of the zinc (II)-cysteine thiolate clusters**

Figure 3. Results of a molecular modeling calculation for rabbit liver $Zn_7$-MT 2A The connectivities for the 7 zinc atoms was determined by NMR studies[12]. The structure of the assembled molecule was calculated using a cycle of molecular mechanics (MM2) and molecular dynamics that searched for minima unconstrained by higher energy conformations of the backbone. The sulfur-zinc bond lengths were determined from XAFS data of Jiang et al.[14]. Reproduced with permission from ref. 7.

Electrospray mass spectrometry offers a new method by which the molar mass of metallothionein, together with its complement of metals, can be determined with precision. Information on the distribution of isoforms and subisoforms is uniquely provided by the mass spectral patterns[33]. Figure 4 shows the mass spectral pattern obtained for the rabbit liver protein used in these studies, isoform 2A, with the pH adjusted to 2.5 with formic acid. Under these conditions the protein's mass is determined for the protonated peptide with no metals bound[33]. The envelope of protonated species shown in Figure 4 encompasses charges of 3+ to 6+ over a m/z range of 1595.4 to 1021.7, respectively. Calculation using the PE-Sciex BioMultiView program provided the mass of the major parent molecule as 6124.0. This corresponds to the fully protonated isoform 2A, 6104+20 $H^+$. We can assign the species responsible for the three higher mass peaks as $Zn_1H_{18}$-MT 2A at 6187.0, and $Zn_4H_{14}$-MT 2A at 6379.0. The peak at 6240.0 is undetermined. Fragmentation of long chain peptides occurs in the inlet manifold so that masses at m/z of 973.6 and 1168.0 can be related to those partial chains.

### SPECTROSCOPIC PROPERTIES: PROBING THE METAL-BINDING REACTION

Two strategies have been used when studying the metal binding reactions of metallothioneins in vitro:

(i) Metals can bind directly and readily to the metal-free or apo-protein.

(ii) Metals with greater binding constants than Zn(II) can displace the Zn(II) in $Zn_7$-MT

Because the Zn(II) in $Zn_7$-MT is tetrahedrally coordinated by the thiolate groups, considerable reorganization must occur in the binding site to accommodate Cu(I) and Ag(I), metals that generally exhibit trigonal or digonal coordination geometries. Metallothioneins containing Ag(I) and Cu(I) exhibit characteristic signatures in their XAFS, circular dichroism, absorption and emission spectra[6].

### METAL BINDING IS REMARKABLE IN MT

Metallothionein is unique amongst metalloproteins in the number of metals bound and the complexity of the metal binding structures. For the metals studied to date, it seems that the clustered description from analysis of the X-ray and NMR data fits as a model that can account for the stoichiometric ratios for the metals not studied by these techniques[1-6]. Optical studies can provide exact values for the numbers of metal atoms bound, and when used with circular dichroism and emission spectroscopic techniques, these numbers can be interpreted in terms of defined structures[6]. Cu(I) binds in a series of steps that suggest that structures form that include three-dimensionally distinct clusters involving 9 Cu(I), 12 Cu(I) and 15 Cu(I) per 20 cysteines, Figure 5[17]. The molar ratio of Cu(I):MT in the best known species of mammalian metallothioneins the $Cu_{12}$-MT[2-6] is associated with $M_6S_9$ (β) and $M_6S_{11}$ (α) clustered domains. Calculated models for these domains are shown in Figures 13 and 14. The stoichiometries for Ag(I) binding to metallothioneins are not as well defined as for Cu(I)[27] as shown in Figures 7, 8 and 9. Finally, Hg(II) and Ag(I) bind with stoichiometries of 18 metals to one protein molecule[6,24,29].

Knowledge of the stoichiometric ratio between the bound metals and the number of accessible cysteinyl sulfurs (Scys) plays a critical role in our understanding of the chemistry, biochemistry, and physiological chemistry of this exceptional protein. The structures formed will depend on the number of metals bound and the coordination preference of the metal. The cross-linking that occurs throughout the peptide chain when 7, 12 or 18 metals are bound depends on both the formation of Scys-M-Scys bonds and on the extent of bridging in which a single cysteine connects two metals. Whether the metals adopt digonal (2-), trigonal (3-) or tetrahedral (4-) coordination dramatically changes the orientation of the peptide chain, resulting in formation of metal-dependent, three-dimensional metal binding sites enclosed by the hydrophilic envelope of the peptide chain.

Spectroscopic signal maxima are observed at metal to protein molar ratios of[1-6]:

- 7, for Hg(II), Cd(II), Co(II), and Fe(II)

- 12, for Cu(I) and Ag(I)

- 18, for Ag(I) and Hg(II).

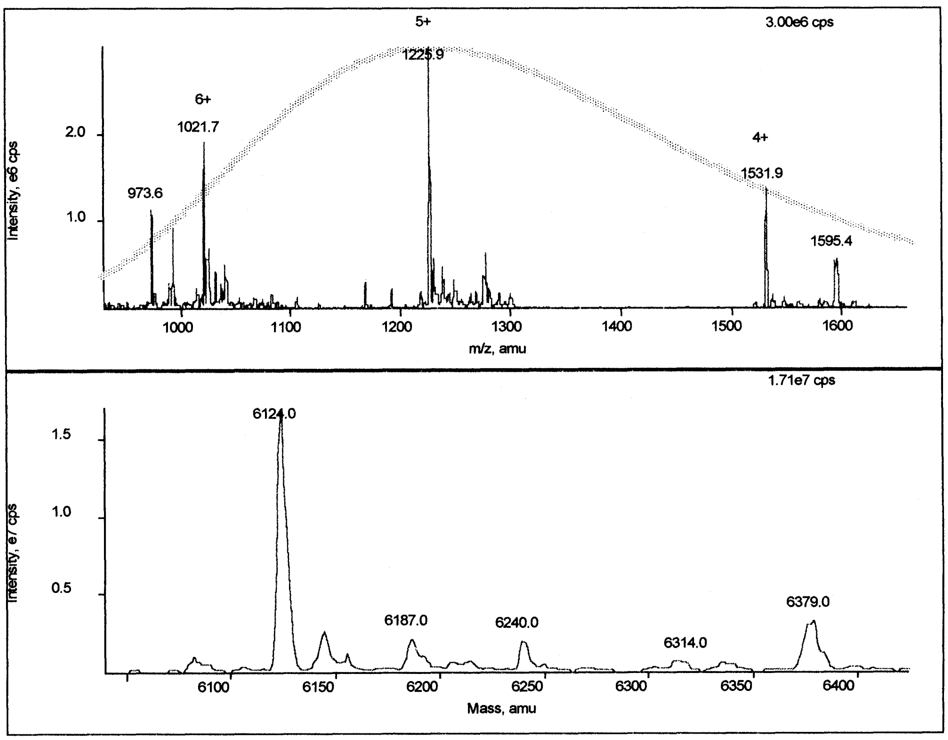

Figure 4 Electrospray ionization mass spectrometry data for a solution of rabbit liver MT 2A adjusted to pH 2.5 with 0.1% formic acid. (A) Shows the mass spectral pattern of the 5 prominent ions, the 4+, 5+, and 6+, that have as a parent molecule the species observed at a mass of 6125 amu. The species with masses at 6146, 6155, and 6188 are from isoforms of the protein.

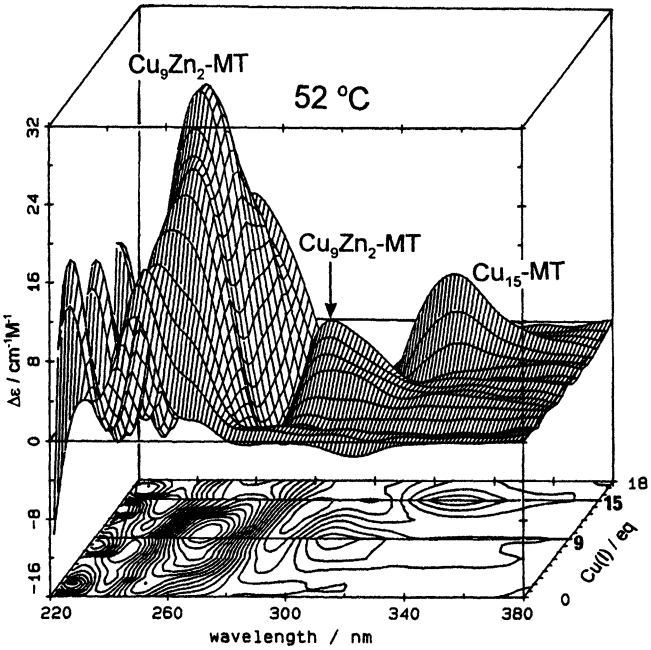

Figure 5 Changes in the CD spectrum of $Zn_7$-MT 2 as Cu(I) is added in stoichiometric ratios of 1 Cu(I) per 20 cysteines at 52 °C. Similar, but less well defined features are observed at lower temperatures. The CD spectrum steeply intensifies and the band maximum red shifts up to the 9 Cu(I) point. The CD spectrum then diminishes as $Cu_{15}$-MT forms with a band maximum near 335 nm. The contour lines show the two maxima that form at the 9 Cu(I) point, and then the red-shifted band that forms for $Cu_{15}$-MT near 340 nm. Reproduced with permission from ref. 17.

### CU(I) AND AG(I) BINDING TO MAMMALIAN MT

While Cu(I) and Ag(I) sometimes exhibit a similar co-ordination chemistry, the CD and emission spectra shown here argue for different structures within the metallothionein binding sites when the metals bind to the rabbit liver protein. First we examine examples of spectral data recorded for copper-containing metallothionein. CD spectra recorded during titrations of $Zn_7$-MT 2 with Cu(I) show the formation of at least 3 major species depending on the temperature. At low temperatures (less than 35 °C, band maxima are observed for molar ratios of 12 and 15 coppers added[17]. However, at elevated temperatures, above 35 °C, the strongest CD signal is recorded for a new species $Cu_9Zn_2$-MT; the data shown in Figure 5 are for a titration carried out at 52 °C. Following the formation of this species, addition of further Cu(I) results in formation of $Cu_{15}$-MT. These striking changes in the CD spectrum are interpreted[17] in terms of a change in the co-ordination geometry around the Cu(I) atom. At low Cu to MT molar ratios, the Cu(I) replaces the tetrahedrally-coordinated Zn(II) with trigonal coordination, up to the 12 Cu(I):20 SH point, see the structure proposed by Presta et al.[17,34], Figures 13 and 14. This structure is represented by a maximum in the CD and emission spectra, Figure 6, at 40 °C. At these high temperatures the peptide chain is able to reorient to accommodate a different wrapping geometry that is thermodynamically preferred for the mixture of 9 trigonally directing Cu(I) atoms and 2 tetrahedrally directing Zn(II) atoms. The co-ordination geometry in $Cu_{15}$-MT undoubtedly includes both digonal and trigonal Cu(I) atoms as the band at 340 nm is not seen below the 15 Cu(I) point. The emission spectra shown as Figure 6 illustrate a second characteristic of metal binding to metallothionein: the reaction in which the metals binds to the binding site is complicated. In this figure, the emission intensity for the first 6 Cu(I) added is about 50 times less than the maximum recorded for $Cu_{12}$-MT.

Figure 7 shows the CD spectra recorded for Ag(I) addition to $Zn_7$-MT at pH 7 and 20 °C from the work of Zelazowski and Stillman[25,27]. Quite clearly, a much simpler system is observed with no reversal in the CD intensity as the molar ratio of Ag:MT increases. In particular, there is little indication of formation of a distinct species at the 12 Ag:MT point. The finally saturation point in

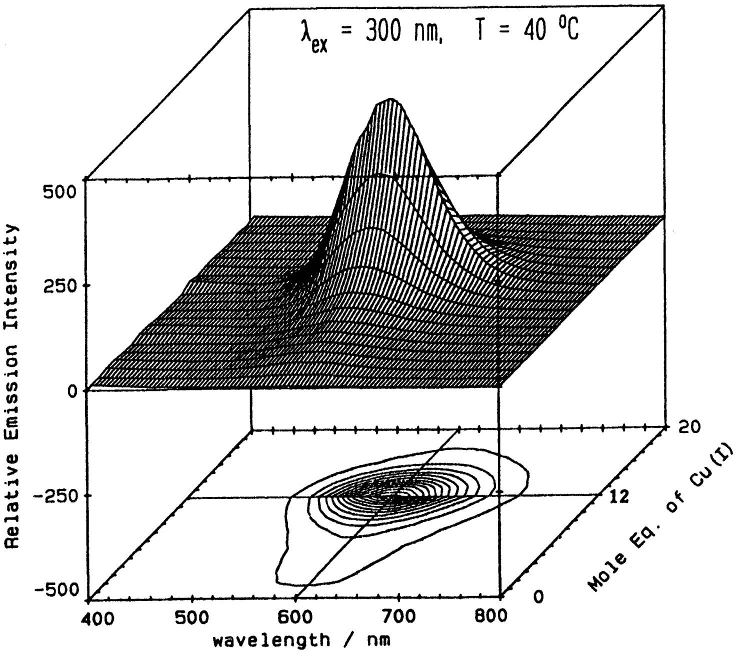

Figure 6 The change in emission spectrum recorded at 40 °C of $Zn_7$-MT 2 as Cu(I) is added in stoichiometric ratios of 1 Cu(I) per 20 cysteines. At these temperatures the emission intensity is extremely low until more than 6 Cu(I) ions have been added. The band maximum at 600 nm occurs for $Cu_{12}$-MT. Reproduced with permission from ref. 21.

the maxima in the CD spectra appear at a ratio of Ag(I) to MT of 18. A similar set of spectra is seen when the metal-free protein is studied[15] at 22 °C, Figure 8. However, now there is indication of species formation at the 12 $Ag^+$ point before the saturation in the signal is observed at the 17 Ag:MT point (isoform 1 saturates at the 17 Ag:MT point compared with the 18 for MT 2A). As in previous studies of Cu(I) binding to MT, we find that different spectral patterns are observed at elevated temperatures. When Ag(I) binds to the apo-MT 1 protein at pH 2.6 and 50 °C, Figure 9, a much more resolved plateau in the CD intensity is observed in the region of 12 Ag(I), strongly suggesting that a structure comparable with that of $Cu_{12}$-MT may be forming. The data suggest that the dominant species is still the $Ag_{17}$-MT 1. Because the spectrum does not include a reversal in sign or significant wavelength shift between 12 and 17, we suggest that the $Ag_{12}$-MT 1 species contains quite similar co-ordination geometry to that in the $Ag_{17}$-MT 1 species. It seems probable that both structures are dominated by digonal co-ordination. Metals bind also to the isolated fragments and exhibit a rich optical spectrum[6]. The CD data for apo-alpha MT as Ag(I) is added[27] exhibit strong features at the 3 Ag:11 S point, which suggests that initially, a non-bridged species forms.

Evidence for the formation of peptides with high mole ratios of Ag(I) is seen in the ES-MS shown as Figure 10[33]. In this figure, a solution with an average of 17 mole equivalents of Ag(I) at pH 2.5 was used. Compared with the ES-MS of the metal-free metallothionein 2A, we see the appearance of a number of new lines in the 7,800 amu region, each representing a peptide with a different number of silver atoms bound. The analysis of the charged species used the envelope of peaks shown in the upper part of Figure 10. Here the 5+ (centered on 1,550 amu) and the 4+ (centered on 1,950 amu) for each of the metallated (Ag = 14 to 19) species can be used to calculate the mass of each parent ion. Under these conditions, the mass spectral data supports the titration data shown in Figures 7, 8 and 9 in which CD maxima for the Ag:MT ratios of 17-18 are observed. The combination of techniques provides solid evidence that these high molecularity species exist as three-dimensional structures as determined by the changes in the CD envelope in the region of the sulfur-to-silver charge transfer bands, rather than adducts, that might be inferred from the mass spectral data alone. On the other hand, the observation of the $Ag_n$-MT species, where n>12, does support the analysis of the CD data as requiring silver binding at mole ratios greater than 12.

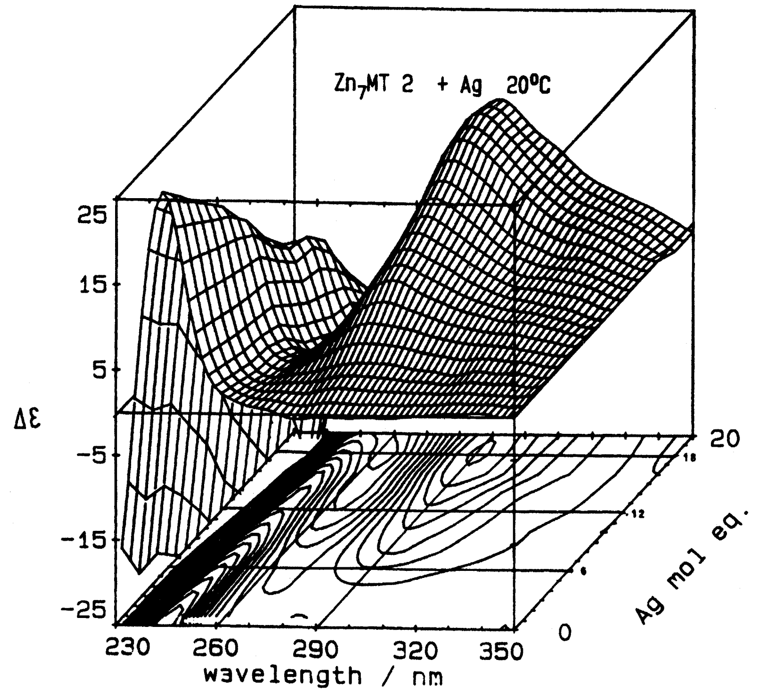

Figure 7 Changes in the CD spectrum of $Zn_7$-MT 2 as Ag(I) is added in stoichiometric ratios of 1 Ag(I) per 20 cysteines at 20 °C. The CD spectrum gradually intensifies with little indication of a band maximum shift up to the 18 Ag(I) point. The CD band near 235 nm due to the Zn-S clusters diminishes as the $Ag_{18}$-MT forms. Reproduced with permission from ref. 25.

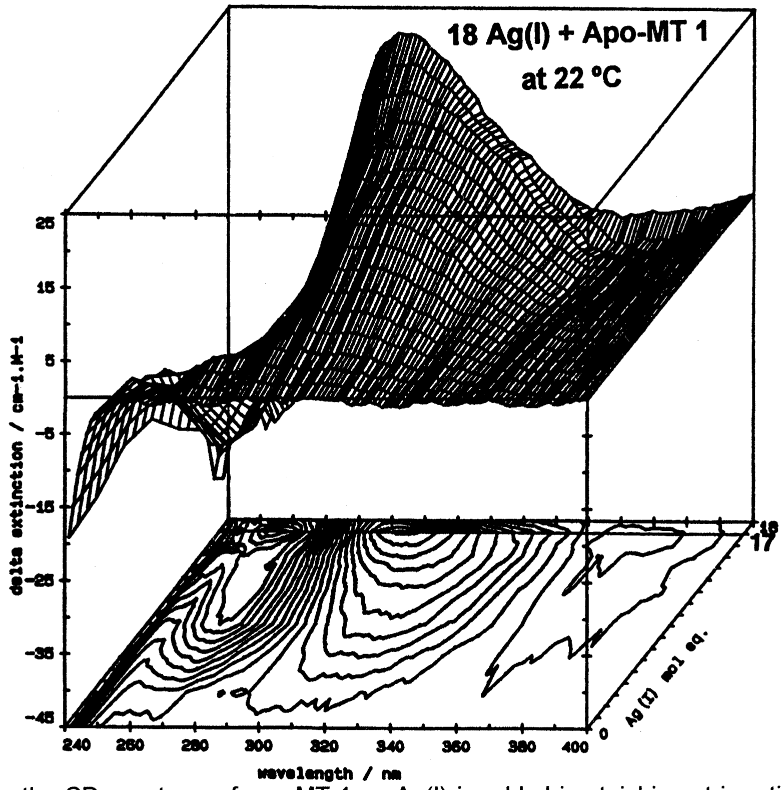

Figure 8 Changes in the CD spectrum of apo-MT 1 as Ag(I) is added in stoichiometric ratios of 1 Ag(I) per 20 cysteines at 22 °C. The CD spectrum gradually intensifies initially with a band maximum in the 270 and 330 nm regions with up to 12 Ag(I) added, then a new blue-shifted band forms up to the 17 Ag(I) point. Reproduced with permission from ref. 15.

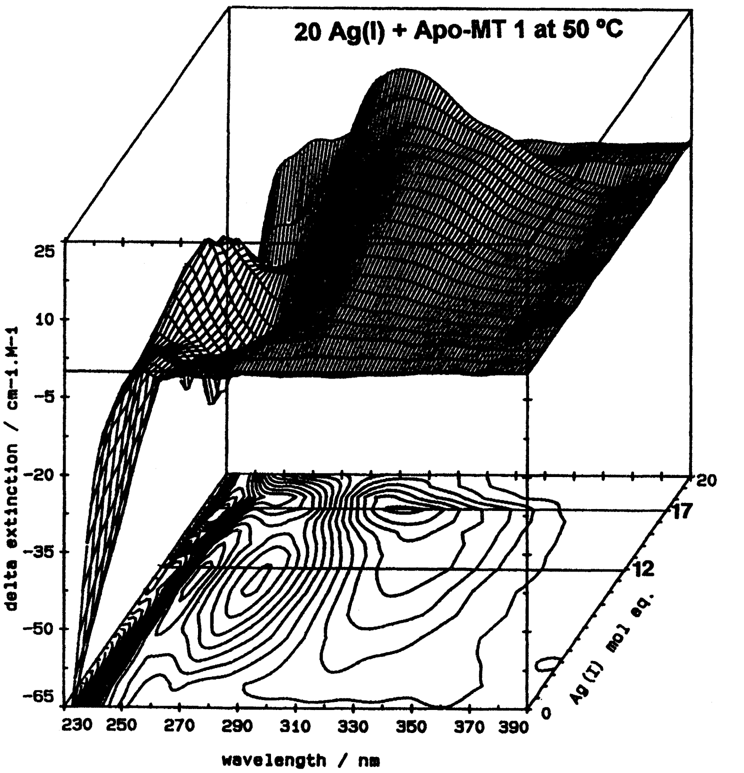

Figure 9 Changes in the CD spectrum of apo-MT 1 as Ag(I) is added in stoichiometric ratios of 1 Ag(I) per 20 cysteines at 50 °C. The CD spectrum gradually intensifies initially with a band maximum in the 270 and 330 nm regions with up to 12 Ag(I) added, then a new blue-shifted band forms up to the 17 Ag(I) point. Reproduced with permission from ref. 15.

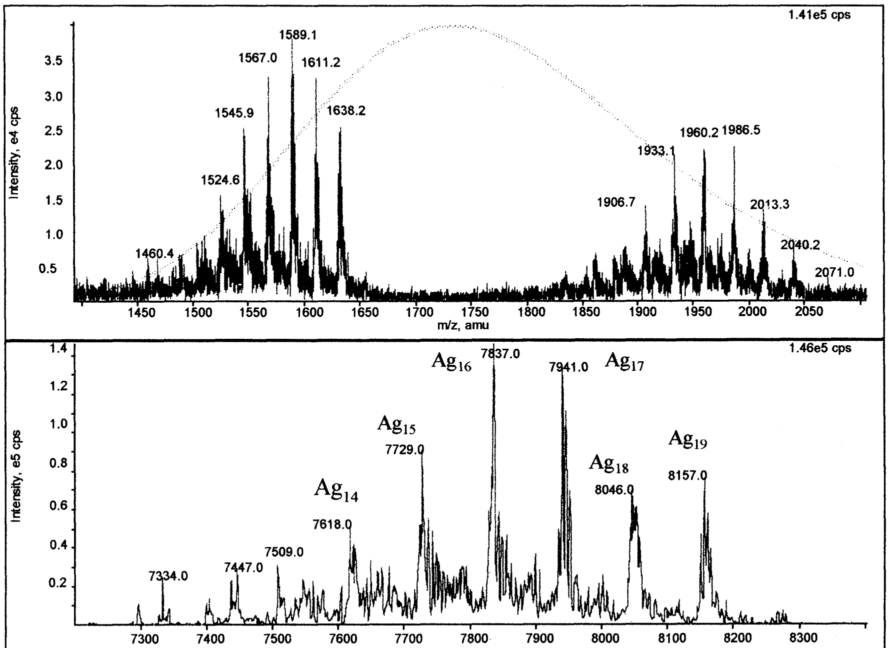

Figure 10 Electrospray mass spectrum for a solution of $Zn_7$-MT 2 at pH 2.5 with 17 mole equivalents of Ag(I) added. The top envelope shows the recorded mass spectrum. The bands at 1589 and 1986, correspond to species with charges of 6+ and 5+. The reconstructed mass spectrum is shown below. The spectrum is very complicated, however, the major peaks can be identified with the following species: $Ag_{14}$-MT, $Ag_{15}$-MT, $Ag_{16}$-MT, $Ag_{17}$-MT, $Ag_{18}$-MT, and $Ag_{19}$-MT. Unpublished results of Stillman, Siu, Guo, Presta.

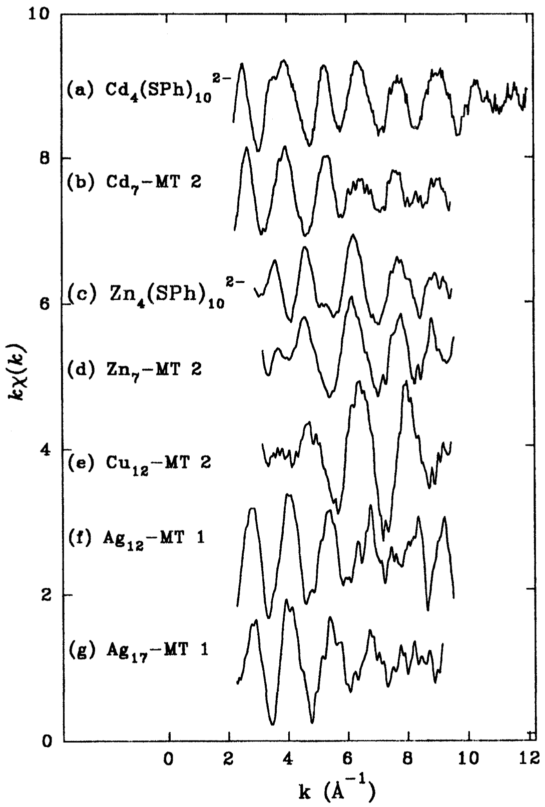

Figure 11 Sulfur-edge XAFS recorded at room temperature for Cd, Zn, Cu and Ag-containing metallothioneins. The data for the two models of the metal-thiolate bridging adopted in the $\alpha$ domain of the metallothionein, $[(Cd_4(SPh)_{10}]^{2-}$ and $[(Zn_4(SPh)_{10}]^{2-}$, show considerable similarities with the data for the proteins. The data for the $Cu_{12}$-MT and $Ag_{12}$-MT species are quite different. Reproduced with permission from ref. 15.

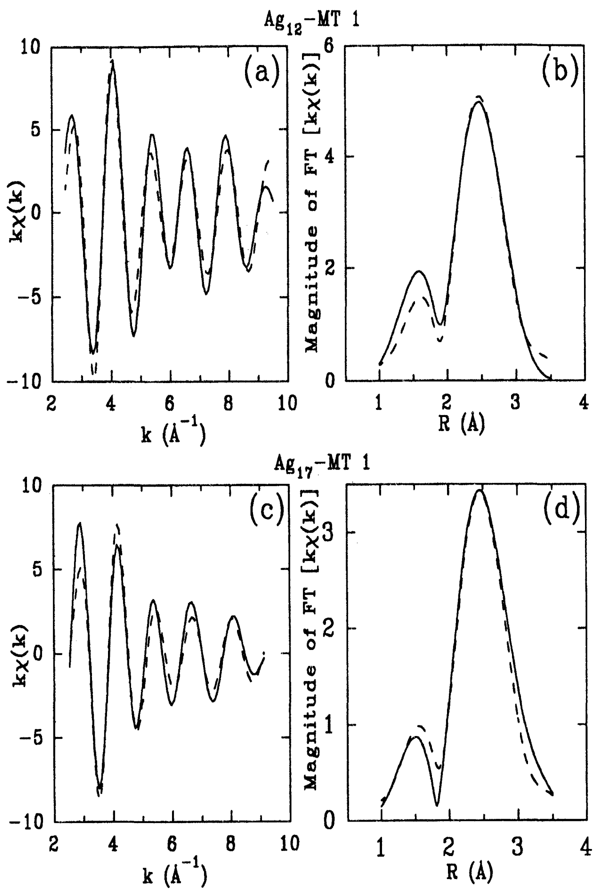

Figure 12 Results of the fitting calculation to the k-space data for $Ag_{12}$-MT 1 and $Ag_{17}$-MT 1. Reproduced with permission from ref. 15.

### THE DYNAMICS OF METAL BINDING

Little is known about the processes that take place as a metal is bound by the metallothionein peptide. One approach that can be used to obtain dynamic data such as this, is to monitor spectroscopic changes that are entirely dependent on the bound metal so that only bound metals contribute to the signal intensity. Changes in the emission spectrum intensity and band maxima wavelengths for data measured that arise directly from the metal binding site have been shown to provide this degree of selectivity for both copper and silver[6]. Figure 6 shows the emission spectral changes recorded as Cu(I) is added to $Zn_7$-MT[21]. In this static experiment the incoming copper ion has had the opportunity to reach equilibrium and, therefore, locate in a thermodynamically stable binding configuration. The data show, however, that the growth in signal intensity is not a linear function of the number of Cu-S bonds that form as would be expected, rather in the early stages the intensity is very low, only past 7 Cu(I) atoms added does the intensity increase in a more linear fashion. This effect is particularly striking at elevated temperatures where the emission intensity for the first 6 Cu(I) added is extremely weak. At low temperatures (<10 $^0$C) a close to linear response is observed from n=1-12. The interpretation of these effects is that the initial Cu(I) binds to the $\beta$ domain which is weakly emissive, possibly because of the greater accessibility of the domain to water. The structural model constructed following energy minimization with molecular mechanics[34] does support this model, Figures 13 and 14.

The time dependence of the kinetic intensity for copper binding also supports a model in which the copper atoms bind equally to the two domains (α and β) but redistributes over a period of 10-30 minutes to populate the β fully to the 6 Cu:MT point. This leads to the formation of the mixed-metal $Cu_6S_9(\beta)Zn_4S_{11}(\alpha)$ MT species[16].

### STRUCTURAL STUDIES

To date, Stout has reported the only protein structure determined from a crystal of $Cd_5Zn_2$-MT from rat liver[8,9]. Other structures have been reported based on metal-based and proton NMR[6,11,12]. Structural information has been obtained for many metals using X-ray absorption techniques (XAS), in particular, XAFS[6,13-15,20,21]. These data provide averages of the metal-sulfur bond lengths and metal co-ordination numbers. Figures 11 and 12 show XAS data for rabbit liver metallothioneins obtained using sulfur-edge XAFS for the model compounds $[Cd_4(SPh)_{10}]^{2-}$ and $[Cd_4(SPh)_{10}]^{2-}$, and the zinc, cadmium, copper and silver-containing protein. The data in Figure 11 illustrate the differences in bond lengths and co-ordination geometries for the three classes of metal-thiolate cluster commonly formed in the mammalian metallothioneins, namely, the $M_7S_{20}$ (Zn and Cd), $M_{12}M_{20}$ (Cu and may be Ag) and $M_{17/18}S_{20}$ (Ag and Hg) species. The analysis of these data is summarized in Table 1. The model compound data for the Cd and Zn proteins provide strong evidence for the analysis in terms of clustered metal-thiolate structures in the protein binding sites. The dissimilarities between the $Cu_{12}$-MT data (e) and the $Ag_{12}$-MT data (f) in Figure 11 provide evidence that the co-ordination geometries in these two protein species are not the same. However, the data in Figure 12 indicate strong similarities between the $Ag_{12}$-MT and the $Ag_{17}$-MT in bond length, Table 1.

Table 1. Summary of structural parameters from analysis of XAFS data of Gui et al. based on sulfur-edge XAFS.[15]

| compound measured | metal-sulfur bond length | | observed CN for the S from analysis of the XAFS data shown in Figure 11 | CN around the metal based on metal XAFS data[6,14] | geometry at the metal |
|---|---|---|---|---|---|
| $Cd_4(SPh)_{10}^{2-}$ | 2.52 ± 0.02 | calculated average co-ordination number for the S based on proposed structure | 1.3 ± 0.4 | 4 | tetrahedral |
| $Cd_7$-MT | 2.54 ± 0.02 | 1.4 | 1.2 ± 0.2 | 4 | tetrahedral |
| $Zn_4(SPh)_{10}^{2-}$ | 2.35 ± 0.03 | 1.6 | 1.5 ± 0.3 | 4 | tetrahedral |
| $Zn_7$-MT | 2.34 ± 0.03 | 1.4 | 1.4 ± 0.3 | 4 | tetrahedral |
| $Cu_{12}$-MT | 2.25 ± 0.01 | 1.8 | 1.7 ± 0.2 | 3[a] | trigonal |
| $Ag_{12}$-MT | 2.45 ± 0.02 | 1.2 | 1.4 ± 0.3 | 2[a] | digonal |
| $Ag_{17}$-MT | 2.44 ± 0.03 | 1.7 | 1.6 ± 0.5 | 2[a] | digonal |

[a]Proposed co-ordination number

Table 2. Summary of the range of metal-sulfur (M = Cu and Ag) bond lengths (Å) in metal-thiolate compounds as a function of co-ordination geometry around the metal[15].

| | Digonal | Trigonal | Tetrahedral |
|---|---|---|---|
| copper(I)-thiolate compounds | 2.15-2.17 | 2.24-2.27 | 2.30-2.42 |
| silver(I)-thiolate compounds | 2.38-2.44 | 2.50-2.58 | 2.55-2.65 |

The sulfur-edge data in Figure 11 can be further analyzed in terms of the extent of bridging sulfur based on the measured co-ordination numbers for the sulfurs. Table 1 shows the results of the analysis for these spectra. The error on the average co-ordination number of the sulfur from the XAFS experiment is very high, so it would be useful to be able to correlate the metal-thiolate bond lengths with co-ordination number, however, as Table 2 shows, the overlap of the ranges of metal-thiolate bond lengths as a function of geometry is much more significant for Ag(I)-S than found for metals such as Zn and Cd. Analysis of the edge data reveal that bridging sulfurs are involved to an increasing degree in the $Ag_{12}$-MT and $Ag_{17}$-MT species. The co-ordination of the sulfur was calculated assuming CN=1 for terminal sulfur and CN=2 for bridging sulfurs in $Ag_{12}S_{20}$ and $Ag_{17}S_{20}$. The observed values shown in Table 1 can be reconciled with model calculations as follows: for $Ag_{12}S_{20}$, a model with 4 bridging and 16 terminal sulfurs gives an average CN of 1.2, whereas for $Ag_{17}S_{20}$, a model with 14 bridging and 6 terminal sulfurs gives an average CN of 1.7. Of course, these calculations do not indicate the structure adopted.

Molecular modeling calculations have been reported for the Zn(II), Cd(II), Hg(II), and Cu(I) containing proteins using the wrapping determined by NMR and X-ray analysis, and bond lengths from XAFS measurements[6,14,15]. Figure 3 shows the results of the calculation for $Zn_7$-MT 2A. A model was constructed for $Cu_{12}$-MT 2A[34] based on the bond lengths and co-ordination numbers from XAS experiments[6,15] and analysis of the Cu(I) binding to $Zn_7$-MT for evidence that the peptide chain remains in the same configuration as the Cu(I) replaces Zn(II). From this analysis, Presta et al.[34] determined that the most likely copper-thiolate structure was that shown in Figure 13. The $Cu_6S_9$ β domain involves bridging for each of the cysteinyl sulfurs forming a hollow, barrel-like

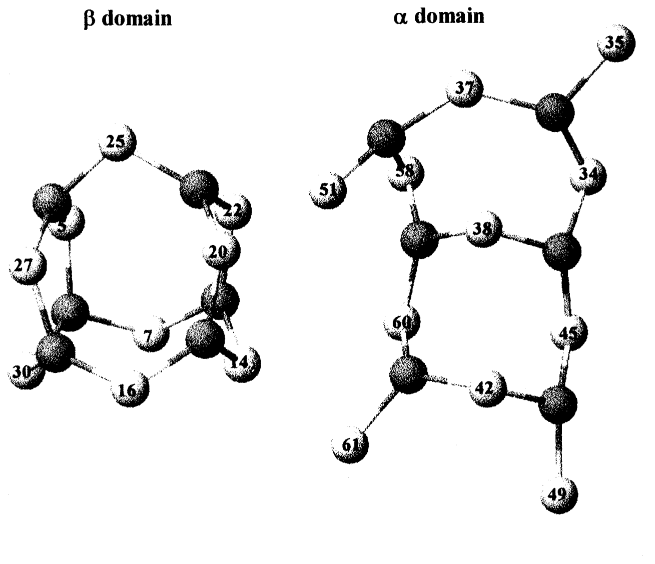

Figure 13 Proposed structure of the copper-thiolate clusters in mammalian metallothioneins based on stoichiometries of $Cu_6S_9$ (beta) and $Cu_6S_{11}$ (alpha) calculated using molecular modeling techniques described for Figures 2 and 3. The Cu(I)-S connectivities were proposed by Presta et al., based on the changes observed in the CD spectrum when Cu(I) replaced Zn(II), see Figure 5. Reproduced with permission from ref. 34.

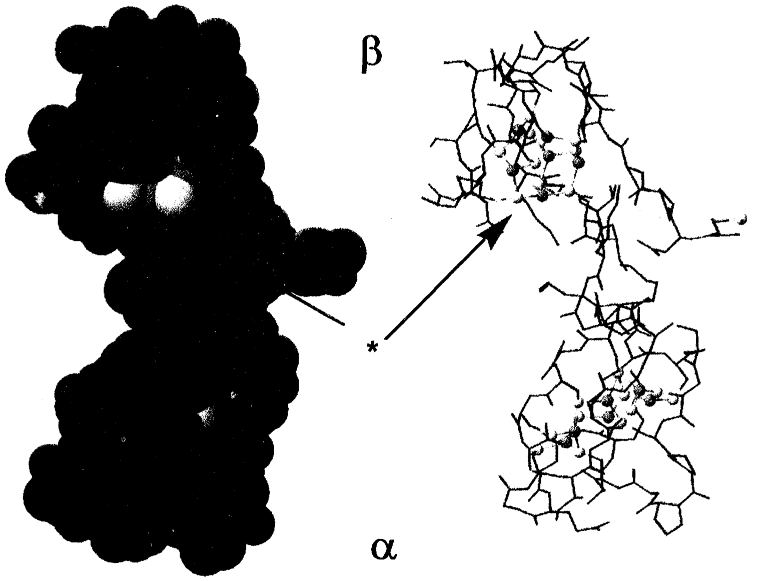

*** Denotes accessible sites of the copper(I)-cysteine thiolate clusters**

Figure 14 Proposed structure of $Cu_{12}$-MT 2 calculated using molecular modeling techniques described in Figure 3. The $Cu_6S_9$ and $Cu_6S_{11}$ domains are almost buried inside the protein in this proposed structure. Reproduced with permission from ref. 34.

Figure 13 Proposed structure of the copper-thiolate clusters in mammalian metallothioneins based on stoichiometries of $Cu_6S_9$ (beta) and $Cu_6S_{11}$ (alpha) calculated using molecular modeling techniques described for Figures 2 and 3. The Cu(I)-S connectivities were proposed by Presta et al., based on the changes observed in the CD spectrum when Cu(I) replaced Zn(II), see Figure 5. Reproduced with permission from ref. 34. structure. The $Cu_6S_{11}$ $\alpha$ domain adopts a ladder-like structure with a mixture of 4 terminal and 7 bridging sulfurs. The three-dimensional structure shown in Figure 14 shows how the peptide envelops the clusters, leaving a single channel or crevice for access in each domain. Compared with the structure of the $Zn_7$-MT shown in Figure 3, we find that the methionine swings down to occupy a region near the lys-lys linker region. The difference in emissive intensities between the $\alpha$ and $\beta$ domains has been accounted for by considering the water access of the two structures. The barrel-like $\beta$ domain appears much more open and, therefore, accessible to water that can readily deactivate the triplet excited state responsible for the emission[6,16,21,34]. Work is in progress on a similar analysis for the $Ag_n$-MT species.

### ACKNOWLEDGMENTS

We thank the Natural Sciences and Engineering Research Council of Canada for funding this work. We are pleased to acknowledge our collaborative work with Professor Mike Siu, York University, Ontario, Canada on the mass spectral properties of metallothioneins. We thank Donald Thomas, U. W.O., for help in recording the ESI-MS data and in preparing each of the figures for publication. MJS would like to acknowledge the very enthusiastic work carried by each member of his group that is discussed in this paper.

### REFERENCES

1. Kagi, J.H.R.; Nordberg, M. (eds.) *Metallothionein*, Birkhauser Verlag, Basel (1979).
2. Kagi, J.H.R.; Kojima, Y. (eds.) *Metallothionein II,* Birkhauser Verlag, Basel (1987).
3. Stillman, M.J.; Shaw, C.F.; Suzuki, K.T (eds.) *Metallothioneins*, V.C.H. Publishers, New York (1992).
4. Suzuki, K.T.; Imura, N.; Kimura, M. (eds.) *Metallothionein III,* Birkhauser Verlag, Basel

(1993).

5. Riordan, J.F.; Vallee, B.L. (eds.) *Metallobiochemistry Part B. Metallothionein and Related Molecules. Methods in Enzymology, vol. 205,* Academic Press, New York (1991).
6. Stillman, M. J. Metallothioneins, *Coord. Chem. Review* **144**, 461-571 (1995).
7. Kagi, J.H.R. in *Metallothionein III,* Suzuki, K.T.; Imura, N.; Kimura, M. Eds., Birkhauser Verlag, Basel, pp. 29-55 (1993).
8. Robbins, A.H.; McRee, D.E.; Williamson, M.; Collett, S.A.; Xuong, N.H.; Furey, W.F.; Wang, B.C.; Stout, C.D. *J. Mol. Biol.* **221**, 1269-1293 (1991).
9. Robbins, A.H.; Stout, C.D. in *Metallothioneins*, Stillman, M.J.; Shaw, C.F. III.; Suzuki, K.T. Eds., VCH Publishers, New York, pp. 31-54 (1992).
10. Otvos J.D.; Armitage, I.M. *Proc. Natl. Acad. Sci. USA* **77**, 7094-7098 (1980).
11. Zhu, Z.; DeRose, E.F.; Mullen, G.P.; Petering, D.H.; Shaw, C.F. III. *Biochem.* **33**, 8858-8865 (1994).
12. Braun, W.; Vasak, M.; Robbins, A.H.; Stout, C.D.; Wagner, G.; Kagi, J.H.R.; Wuthrich, K. *Proc. Natl. Acad. Sci. U.S.A.* **89**, 10124-10128 (1992).
13. George, G.N.; Byrd, J.; Winge D.R *J. Biol. Chem.* **263**, 8199-8203 (1988).
14. Jiang, D.T.; Gui, Z.Q.; Heald, S.M.; Sham, T.K.; Stillman, M.J. *Physica B* **208/209**, 729-730 (1995).
15. Gui, Z.; Green, A.R.; Kasrai, M.; Bancroft, G.M.; Stillman, M.J. *Inorganic Chemistry,* **35**, 6520-6529 (1996).
16. Green, A.R.; Stillman, M.J. *Inorg. Chem.* **35**, 2799-2807 (1996).
17. Presta, A.; Green, A.R.; Zelazowski, A.J.; Stillman, M.J. *Eur. J. Biochem.* **227**, 226-240 (1995).
18. Presta, A.; Green, A.R.; Stillman, M.J. (1999) submitted.
19. Stillman, M.J.; Presta, A.; Gui, Z.; Jiang, D.T. *Metal-Based Drugs* **1**, 375-393 (1994).
20. Jiang, D.T.; Heald, S.M.; Sham, T.K.; Stillman, M.J. *J. Am. Chem. Soc.* **116**, 11004-11013 (1994).
21. Green, A.R.; Presta, A,; Gasyna, Z; Stillman, M.J. *Inorg. Chem.* **33**, 4159-4168 (1994).
22. Presta, A.; Stillman, M.J. *Chirality* **6**, 521-530 (1994).
23. Lu, W.; Stillman, M.J. *J. Am. Chem. Soc.* **115**, 3291-3299 (1993).
24. Lu, W.; Zelazowski, A.J.; Stillman, M.J. *Inorg. Chem.* **32**, 919-926 (1993).
25. Zelazowski, A.J.; Stillman, M.J. *Inorg. Chem.* **31**, 3363-3370 (1992).
26. Lu, W.; Kasrai, M.; Bancroft, G.M.; Stillman, M.J.; Tan, K.H. *Inorg. Chem.* **29**, 2561-2563 (1990).
27. Zelazowski, A.J.; Gasyna, Z.; Stillman, M.J. *J. Biol. Chem.* **264**, 17091-17099 (1989).
28. Stillman, M.J.; Zelazowski, A.J.; Gasyna, Z. *FEBS Lett.* **240**, 159-162 (1988).
29. Cai, W.; Stillman, M.J. *J. Am. Chem. Soc.* **110,** 7872-7873 (1988).
30. Stillman, M. J.; Cai, W.; Zelazowski, A. J. *J. Biol. Chem.* **262**, 4538-4548 (1987).
31. Stillman, M.J.; Zelazowski, A.J. *J. Biol. Chem.* **263**, 6128-6133 (1988)
32. Fowle, D.A.; Stillman, M.J. *J. Biomol. Struct. Dyn.* **14,** 393-406 (1997).
33. Le Blanc, Y.J.C.; Presta, A.; Veinot, J.; Gibson, D.; Siu, K.W.M.;Stillman, M.J. *Protein Peptide Lett.*, **4**, 313-320 (1997).
34 Presta, A.; Fo wle, D. A.;Stillman, M. J. *J. Chem. Soc. Dalton Trans.* 977-984 (1997).

